# Association of plasma levels of Sestrin2 with adiposity and metabolic function indices in healthy and diabetic subjects from Qatar Biobank

**DOI:** 10.3389/fendo.2025.1518388

**Published:** 2025-05-13

**Authors:** Muhammad Ammar Zahid, Shahenda Salah Abdelsalam, Hicham Raïq, Hanan H. Abunada, Aijaz Parray, Abdelali Agouni

**Affiliations:** ^1^ Department of Pharmaceutical Sciences, College of Pharmacy, QU Health, Qatar University, Doha, Qatar; ^2^ Department of Social Sciences, College of Arts and Sciences, Qatar University, Doha, Qatar; ^3^ Office of Vice President for Medical and Health Sciences, QU Health, Qatar University, Doha, Qatar; ^4^ The Neuroscience Institute, Academic Health System, Hamad Medical Corporation, Doha, Qatar

**Keywords:** adiposity, biomarker, diabetes mellitus, metabolic syndrome, Sestrin2

## Abstract

**Background:**

Despite the accumulating evidence from cellular and animal studies, the role of circulating Sestrin2, a stress-inducible antioxidant protein, in human cardiometabolic health remains largely unexplored. Hence, the current study aimed to investigate the association between circulating Sestrin2 and cardiometabolic risk factors in healthy and diabetic individuals.

**Methods:**

This cross-sectional study leveraging data and plasma samples from the Qatar Biobank investigated the relationship between plasma Sestrin2 levels and various cardiometabolic indices in 326 healthy and 518 diabetic subjects.

**Results:**

The study found that Sestrin2 levels were significantly lower in diabetic individuals compared to healthy controls (5.49 ng/mL *vs* 8.25 ng/mL, p < 0.001). In the healthy cohort, higher Sestrin2 levels were associated with a favorable metabolic profile, indicated by lower odd ratios (OR) of high glycated hemoglobin (OR: 0.33), Homeostatic Model Assessment for Insulin Resistance score (OR: 0.58), visceral adiposity index (OR: 0.46), lipid accumulation product (OR: 0.49), atherogenic index of plasma (OR: 0.42) and metabolic syndrome (OR: 0.23). Conversely, in the diabetic cohort, higher Sestrin2 levels were paradoxically linked to increased triglycerides (OR: 1.57), the product of triglyceride glucose and waist circumference (OR: 1.8), body fat (OR: 1.72), waist circumference (OR: 1.82), waist-to-hip ratio (OR: 1.96) and metabolic syndrome (OR: 1.48).

**Conclusions:**

These findings suggest that Sestrin2 may play a complex and context-dependent role in metabolic regulation, potentially serving as a protective factor in healthy individuals but contributing to metabolic dysfunction in the context of established diabetes. Further research is needed to elucidate the underlying mechanisms and implications for targeted interventions.

## 1 Introduction

Cardiovascular diseases, such as coronary heart disease and stroke, and metabolic disorders, including type 2 diabetes and obesity, are closely interlinked and pose a significant challenge to global health and economic stability. Diabetes is identified as a principal risk factor for the onset and advancement of cardiometabolic diseases (1). The intricate relationship between diabetes and cardiometabolic dysfunction necessitates immediate efforts to elucidate the underlying molecular mechanisms. Such efforts could facilitate the identification of novel therapeutic strategies and diagnostic biomarkers for early detection, thereby mitigating the impact of these diseases (2). Sestrins, a family of stress-responsive proteins, have recently emerged as significant players in metabolic regulation. This group, consisting of Sestrin1 (SESN1), Sestrin2 (SESN2), and Sestrin3 (SESN3), is involved in various cellular processes, including antioxidant defense, autophagy, and metabolic equilibrium (3, 4). SESN2, in particular, has attracted attention due to its comprehensive functions and potential therapeutic implications (5). It has been implicated in numerous signaling pathways that govern glucose and lipid metabolism (6). Research in cellular and animal models of metabolic disruption indicates that SESN2 deficiency results in impaired glucose tolerance, insulin resistance, and hepatic steatosis, underscoring its critical role in metabolic balance (7). Conversely, the overexpression or activation of SESN2 has been shown to counteract metabolic dysfunction and protect against diet-induced obesity and insulin resistance by modulating AMP-activated protein kinase (AMPK) and mammalian target of rapamycin (mTOR) pathways (8). These findings position SESN2 as a pivotal regulator of metabolic health and a promising target for therapeutic intervention in cardiometabolic diseases.

Several studies have shown that SESN2 protein is detectable in human plasm and serum using ELISA-based assays. Although the exact source of SESN2 in circulation is not fully clear, numerous studies have proposed that immune cells are the contributors of SESN2 in circulation (9–11). Despite the accumulating evidence from cellular and animal studies, the role of circulating SESN2 in human cardiometabolic health remains largely unexplored. While certain studies have examined the association between *SESN2* gene expression and metabolic parameters in specific populations (12), the link between circulating SESN2 levels and cardiometabolic risk factors, especially in the context of diabetes, is not well understood. Recent investigations have revealed a positive correlation between plasma concentrations of SESN2 and cardiometabolic disturbances in diabetic patients, suggesting a protective role for SESN2 in diabetes and its related complications (13, 14). However, in the context of diabetic complications, SESN2 levels were observed to decrease compared to levels in diabetes alone, indicating disease progression (15).

Given the evidence that circulating levels of SESN2 are associated with diabetes and its complications, yet acknowledging the complexity of this relationship influenced by various factors, further research is crucial to clarify the clinical significance of SESN2 in human metabolic health and disease. This study aims to address this gap by examining the association between plasma SESN2 levels and various adiposity and metabolic function indices in both healthy individuals and diabetic patients. Utilizing the extensive resources of the Qatar Biobank (QBB), this research seeks to elucidate the potential role of SESN2 as a biomarker of cardiometabolic health.

The relevance of this study extends to the specific demographic of Qatar, where diabetes and cardiometabolic diseases are highly prevalent, representing a substantial public health concern (16, 17). Investigating SESN2’s role within the Qatari population could provide valuable insights into the unique genetic and environmental factors influencing these diseases’ development and progression in this context. Such knowledge could inform targeted prevention and treatment strategies tailored to the Qatari population’s needs.

## 2 Materials and methods

### 2.1 Ethical approval

The study protocol was approved by the Institutional Review Boards (IRB) of Qatar Biobank (QBB) (#Ex-2021-QF-QBB-RES-ACC-00049-0173*)* and Qatar University (#QU-IRB 1624-E/21). Furthermore, the study received approval from the Qatar University Institutional Biohazard Committee (IBC) (#QU-IBC-2021/046). All participants provided informed consent before data collection. Data provided by Qatar Biobank was anonymized to protect participant confidentiality.

### 2.2 Study design and participants

This cross-sectional study used data and samples from the QBB, which is a prospective, population-based study initiated in 2012 and aims to collect health data and biological samples for research purposes (18). The QBB cohort aims to recruit 60,000 adult Qataris or long-term residents (adults for ≥ 15 years in Qatar) and follow them up to 5 years (18). The study population consisted of two cohorts, a healthy group (N=326) and a diabetes mellitus group (N=518) as defined by the International Diabetes Federation (IDF) criteria (19). Inclusion criteria were adult participants from the QBB biorepository (aged ≥18 years) with data on the cardiometabolic parameters and availability of plasma to measure SESN2 protein levels. Exclusion criteria included the comorbidities that may interfere with the results, such as chronic infections, severe inflammatory conditions, cancer, and pregnant women. The healthy control subjects taking any medications at the time of enrollment were also excluded.

### 2.3 Data collection

Demographic data, such as participants’ questionnaires regarding age, gender, and nationality, was collected from the participants by trained nurses. Anthropometric indicators such as height, weight, waist circumference, and waist-to-hip ratio were measured directly by using equipment from Seca, Hamburg Germany. Blood pressure recordings were an average of three measurements. Blood was withdrawn after overnight fasting and the biochemical parameters, namely glucose, insulin, glycated hemoglobin (HbA1c), lipid profiles, and other such factors were analyzed in the participants by accepted laboratory standards and automated equipment (Hitachi-917, Gmbh Diagnostic, Mannheim, Germany) in the clinical chemistry laboratory of Hamad Medical Corporation (HMC). The secondary indices Homeostatic Model Assessment of Insulin Resistance score (HOMA-IR) (Equation 1), Triglyceride Glucose (TyG) (Equation 2), Visceral Adiposity Index (VAI) (Equations 3, 4), Lipid Accumulation Product (LAP) (Equations 5, 6) and Atherogenic Index of Plasma (AIP) (Equation 7) were calculated from the primary measurements using following equations already reported in the literature (20–24). Metabolic syndrome (MetS) was defined using the IDF criteria of central obesity with two or more of the following four: elevated concentrations of triglycerides, reduced concentrations of High high-density lipoprotein Cholesterol (HDL-C), elevated blood pressure, and dysglycemia (25).


(1) $$\text{HOMA-IR}=\frac{\text{Glucose}(\text{mmol}/\text{L})\times \text{Insulin}(\text{μIU}/\text{mL})}{22.5}$$

(2) $$\text{TyG}=\ln(\frac{\text{Triglycerides}(\text{mg}/\text{dL})\times \text{Glucose}(\text{mg}/\text{dL})}{2})$$

(3) $${\text{VAI}}_{\text{Male}}=\frac{\text{Waist Circumference}(\text{cm})}{39.68+1.88\times \text{BMI}(\text{kg}/{\text{m}}^{2})}\times \frac{\text{Triglycerides}(\text{mmol}/\text{L})}{1.03}\times \frac{1.31}{\text{HDL Cholesterol}(\text{mmol}/\text{L})}$$

(4) $${\text{VAI}}_{\text{Female}}=\frac{\text{Waist Circumference}(\text{cm})}{36.58+1.89\times \text{BMI}(\text{kg}/{\text{m}}^{2})}\times \frac{\text{Triglycerides}(\text{mmol}/\text{L})}{0.81}\times \frac{1.52}{\text{HDL Cholesterol}(\text{mmol}/\text{L})}$$

(5) $${\text{LAP}}_{\text{Male}}=(\text{Waist Circumference}(\text{cm})-65)\times \text{Triglycerides}(\text{mmol}/\text{L})$$

(6) $${\text{LAP}}_{\text{Female}}=(\text{Waist Circumference}(\text{cm})-58)\times \text{Triglycerides}(\text{mmol}/\text{L})$$

(7) $$\text{AIP}=\log_{10}(\frac{\text{Triglycerides}(\text{mmol}/\text{L})}{\text{HDL Cholesterol}(\text{mmol}/\text{L})})$$

### 2.4 SESN2 measurement

Blood samples were collected using EDTA tubes, and plasma was separated using centrifugation at 3,000 RPM for 20 minutes. Aliquots of 250 μL of plasma were stored in the liquid nitrogen vapor phase until further detection. Plasma SESN2 levels were measured using an enzyme-linked immunosorbent assay (ELISA) kit purchased from a commercial firm (SESN2 ELISA Kit, BT LAB, Cat. No. E3437Hu) with a sensitivity of 0.01 ng/mL and detection limit of 0.05-15 ng/mL. Briefly, plasma samples were brought to room temperature, 40 μL of the sample was centrifuged at 3,000 RPM for 5 minutes, and the supernatant was used for the assay. Ten μL of the anti-SESN2 antibody and 50 μL of the streptavidin HRP solution were added into each well and incubated for an hour at 37°C. After washing, substrate solution was added, and the incubation was performed for another 10 minutes. Finally, the reaction was stopped by adding stop solution, and optical density was measured at 450 nm. Initially, all samples were tested without dilution. If SESN2 was found to be beyond the upper detection limit of 15ng/mL, samples were diluted up to 2.5 times to bring the concentration under the detection limit. The intra and inter-assay coefficients of variation were in acceptable ranges of less than 8% and 10%, respectively.

### 2.5 Statistical analysis

All the above data was analyzed using R (Version 4.4). Means and standard deviations (for continuous variables), as well as frequencies and percentages (for categorical variables), were computed as descriptive statistics to summarize the demographic and clinical characteristics of the study population. Independent t-tests or Mann-Whitney U tests, as appropriate, were applied to compare continuous variables within groups such as healthy *vs* diabetic or SESN2 tertiles within each group for healthy and diabetic participants. The Chi-square test was used to compare categorical variables between two or more independent groups (e.g., healthy *vs* diabetic, SESN2 tertiles). Spearman’s correlation coefficients were calculated to examine the linear relationships between SESN2 concentration and various numerical parameters, including metabolic markers, adiposity indices, and blood pressure. To account for potential confounding effects, partial correlations were computed, adjusting for age, gender, and nationality. Logistic regression models were constructed to investigate the associations between SESN2 concentration (as a categorical variable based on tertiles) and binary outcomes, such as high glucose, high HbA1c, and MetS. Odds ratios (ORs) and associated p values were estimated, adjusting for age, gender, and nationality.

## 3 Results

### 3.1 Demographics and clinical characteristics

The demographics and clinical characteristics of the healthy (N = 326) and diabetes subpopulations (N = 518), as well as their comparison, are shown in **Table 1**. The mean age in the healthy cohort was 35.37 years, and more than half of the respondents (53%) were women. The mean BMI was in the overweight category (28.63 kg/m²). Many subjects of the healthy cohort nevertheless showed features of metabolic dysfunction. Namely, 72% of the cohort comprised overweight people, 65% had elevated low-density lipoprotein cholesterol (LDL-C), 69% had a high TyG index, and 56% had a high HOMA-IR score. The diabetic cohort had a mean age of 49.87 years with more males than females, 57%. As anticipated, the diabetic population demonstrated more metabolic dysfunction than the healthy population. This was observable with the higher mean BMI (30.76 kg/m²), high fasting glucose (9.80 mmol/L), and the presence of several components of the MetS. All subjects in this cohort had high HbA1c levels, and 98% of the have a high TyG index while 90% had high HOMA-IR scores. The SESN2 levels in the diabetic cohort were significantly lower than the healthy individuals (5.49 ± 5.94 *vs* 8.25 + 7.57, p < 0.001).

**Table 1:** Baseline characteristics and the comparison of the healthy cohort with the diabetes cohort.

Characteristic	Overall, N = 844	Healthy, N = 326	Diabetes, N = 518	p-value
Gender				0.004
- Female	393 (47%)	172 (53%)	221 (43%)	
- Male	451 (53%)	154 (47%)	297 (57%)	
Age (years)	44.27 ± 13.46	35.37 ± 11.85	49.87 ± 11.20	**<0.001**
BMI (kg/m²)	29.94 ± 5.72	28.63 ± 5.74	30.76 ± 5.56	**<0.001**
Biochemical Measurements				
SESN2 (ng/mL)	6.55 ± 6.75	8.25 ± 7.57	5.49 ± 5.94	**<0.001**
Glucose (mmol/L)	7.89 ± 3.93	4.87 ± 0.67	9.80 ± 3.93	**<0.001**
Insulin (µIU/mL)	19.89 ± 42.17	14.17 ± 15.53	23.54 ± 52.21	**<0.001**
HbA1c (%)	7.22 ± 2.02	5.33 ± 0.38	8.41 ± 1.70	**<0.001**
Total Cholesterol (mmol/L)	4.78 ± 1.00	4.78 ± 0.87	4.79 ± 1.08	0.7
HDL Cholesterol (mmol/L)	1.30 ± 0.36	1.38 ± 0.36	1.25 ± 0.35	**<0.001**
LDL Cholesterol (mmol/L)	2.81 ± 0.89	2.88 ± 0.76	2.77 ± 0.96	**0.018**
Triglyceride (mmol/L)	1.52 ± 0.99	1.15 ± 0.73	1.75 ± 1.06	**<0.001**
Insulin Resistance and Adiposity				
Waist Size (cm)	93.82 ± 14.50	87.45 ± 14.32	97.83 ± 13.12	**<0.001**
Waist-to-Hip Ratio	0.88 ± 0.10	0.82 ± 0.10	0.92 ± 0.09	**<0.001**
HOMA-IR	7.44 ± 16.31	3.31 ± 5.28	10.07 ± 20.01	**<0.001**
TyG Index	8.91 ± 0.81	8.28 ± 0.51	9.31 ± 0.70	**<0.001**
TG/HDL Ratio	1.34 ± 1.15	0.95 ± 0.92	1.58 ± 1.21	**<0.001**
TyG x BMI	267.38 ± 59.36	237.97 ± 53.79	285.95 ± 55.10	**<0.001**
TyG x Waist Circumference	839.73 ± 169.03	727.45 ± 145.18	910.67 ± 142.60	**<0.001**
TyG x Waist-to-Hip Ratio	7.89 ± 1.38	6.83 ± 1.09	8.55 ± 1.11	**<0.001**
Visceral Adiposity Index (VAI)	2.02 ± 1.76	1.39 ± 1.25	2.41 ± 1.91	**<0.001**
Lipid Accumulation Product (LAP)	51.20 ± 40.29	33.06 ± 28.51	62.65 ± 42.40	**<0.001**
Atherogenic Index of Plasma (AIP)	0.02 ± 0.30	-0.12 ± 0.27	0.10 ± 0.29	**<0.001**
Body Fat Percentage (%)	31.99 ± 10.93	31.10 ± 11.60	32.57 ± 10.45	0.090
Blood Pressure				
Average Systolic BP (mmHg)	119.32 ± 14.85	112.69 ± 12.62	123.50 ± 14.63	**<0.001**
Average Diastolic BP (mmHg)	69.58 ± 10.18	67.31 ± 9.84	71.01 ± 10.15	**<0.001**
**Metabolic Syndrome (MetS)**	355 (42%)	55 (17%)	300 (58%)	**<0.001**
Condition1 (Central obesity)	594 (70%)	184 (56%)	410 (79%)	**<0.001**
Condition2 (High Triglyceride)	253 (30%)	45 (14%)	208 (40%)	**<0.001**
Condition3 (Low HDL)	293 (35%)	87 (27%)	206 (40%)	**<0.001**
Condition4 (High Glucose)	608 (72%)	90 (28%)	518 (100%)	**<0.001**
Condition5 (High BP)	204 (24%)	37 (11%)	167 (32%)	**<0.001**

Values in bold indicate statistically significant results (p < 0.05).

### 3.2 Comparison among tertile groups

The healthy cohort (N = 326) was stratified into three groups based on SESN2 concentration tertiles (T1: lowest, T3: highest) as shown in **Table 2**. As expected, a significant difference in SESN2 concentration was observed across tertiles (p <0.001), validating the grouping strategy. Interestingly, insulin levels (p = 0.005) and HbA1c (p = 0.007) were significantly higher in the lowest SESN2 tertile (T1) compared to the higher tertiles. This suggests a potential link between lower SESN2 levels and impaired insulin sensitivity even within a healthy population. The HOMA-IR score, an indicator of insulin resistance, was higher in T1 as compared to T2 and T3 (p = 0.017). While not statistically significant, there was a trend towards higher triglycerides and lower HDL-C in the lowest SESN2 tertile. This further supports the notion that lower SESN2 might be associated with a less favorable metabolic profile. Additionally, there was a higher prevalence of MetS (p<0.001) and several of its components in T1, suggesting that lower SESN2 might contribute to a clustering of cardiometabolic risk factors.

**Table 2:** Comparative analysis of cardiometabolic indices across tertile groups of SESN2 in the healthy cohort.

Characteristic	Overall, N = 326	T1, N = 109	T2, N = 108	T3, N = 109	p-value
Gender					0.4
- Female	172 (53%)	54 (50%)	55 (51%)	63 (58%)	
- Male	154 (47%)	55 (50%)	53 (49%)	46 (42%)	
Age (years)	35.37 ± 11.85	36.14 ± 12.73	34.82 ± 12.69	35.14 ± 10.01	0.6
BMI (kg/m²)	28.63 ± 5.74	29.25 ± 6.13	28.06 ± 5.66	28.57 ± 5.42	0.5
Biochemical Measurements					
SESN2 Concentration (ng/mL)	8.25 ± 7.57	2.49 ± 1.29	5.26 ± 1.02	16.98 ± 7.11	**<0.001**
Glucose (mmol/L)	4.87 ± 0.67	4.85 ± 0.62	4.82 ± 0.55	4.92 ± 0.80	0.9
Insulin (µIU/mL)	14.17 ± 15.53	16.36 ± 13.65	13.18 ± 11.07	12.95 ± 20.25	**0.005**
HbA1c (%)	5.33 ± 0.38	5.43 ± 0.38	5.29 ± 0.40	5.28 ± 0.35	**0.007**
Total Cholesterol (mmol/L)	4.78 ± 0.87	4.71 ± 0.86	4.78 ± 0.88	4.85 ± 0.87	0.5
HDL Cholesterol (mmol/L)	1.38 ± 0.36	1.35 ± 0.34	1.39 ± 0.38	1.41 ± 0.37	0.5
LDL Cholesterol (mmol/L)	2.88 ± 0.76	2.80 ± 0.78	2.87 ± 0.74	2.97 ± 0.76	0.3
Triglyceride (mmol/L)	1.15 ± 0.73	1.24 ± 0.77	1.18 ± 0.90	1.04 ± 0.44	0.4
Insulin Resistance and Adiposity					
Waist Size (cm)	87.45 ± 14.32	89.86 ± 15.59	85.57 ± 13.85	86.91 ± 13.22	0.13
Waist-to-Hip Ratio	0.82 ± 0.10	0.84 ± 0.11	0.82 ± 0.09	0.81 ± 0.10	0.2
HOMA-IR	3.31 ± 5.28	3.75 ± 3.82	2.92 ± 2.80	3.25 ± 7.80	**0.017**
TyG Index	8.28 ± 0.51	8.33 ± 0.58	8.28 ± 0.52	8.23 ± 0.43	0.6
TG/HDL Ratio	0.95 ± 0.92	1.06 ± 0.83	0.99 ± 1.26	0.82 ± 0.50	0.3
TyG x BMI	237.97 ± 53.79	244.84 ± 58.68	233.28 ± 52.21	235.76 ± 49.88	0.4
TyG x Waist Circumference	727.45 ± 145.18	753.02 ± 163.62	711.62 ± 137.25	717.57 ± 130.33	0.2
TyG x Waist-to-Hip Ratio	6.83 ± 1.09	7.01 ± 1.22	6.77 ± 1.03	6.72 ± 1.00	0.2
Visceral Adiposity Index (VAI)	1.39 ± 1.25	1.54 ± 1.18	1.43 ± 1.67	1.21 ± 0.70	0.4
Lipid Accumulation Product (LAP)	33.06 ± 28.51	39.34 ± 33.87	30.73 ± 27.91	29.08 ± 21.60	0.2
Atherogenic Index of Plasma (AIP)	-0.12 ± 0.27	-0.08 ± 0.30	-0.11 ± 0.27	-0.15 ± 0.23	0.3
Body Fat Percentage (%)	31.10 ± 11.60	32.05 ± 12.42	29.60 ± 11.33	31.64 ± 10.98	0.3
Blood Pressure (BP)					
Average Systolic BP (mmHg)	112.69 ± 12.62	114.50 ± 14.06	111.91 ± 12.63	111.65 ± 10.89	0.3
Average Diastolic BP (mmHg)	67.31 ± 9.84	67.98 ± 10.85	65.84 ± 9.31	68.10 ± 9.18	0.2
**Metabolic Syndrome (MetS)**	55 (17%)	33 (30%)	12 (11%)	10 (9.2%)	**<0.001**
Condition1 (Central obesity)	184 (56%)	65 (60%)	57 (53%)	62 (57%)	0.6
Condition2 (High Triglyceride)	45 (14%)	21 (19%)	16 (15%)	8 (7.3%)	**0.036**
Condition3 (Low HDL)	87 (27%)	30 (28%)	27 (25%)	30 (28%)	0.9
Condition4 (High Glucose)	90 (28%)	39 (36%)	26 (24%)	25 (23%)	0.064
Condition5 (High BP)	37 (11%)	19 (17%)	11 (10%)	7 (6.4%)	**0.034**

Values in bold indicate statistically significant results (p < 0.05).

Like the healthy cohort, the diabetic cohort (N = 518) was stratified into SESN2 tertiles (**Table 3**). Significant differences in SESN2 concentration were observed across tertiles (p <0.001). The insulin levels were higher in lower tertile T1 (p = 0.008), but unlike the healthy cohort, HBA1c did not show a statistically significant pattern across tertiles in the diabetic group and the HOMA-IR score was high in the highest tertile (T3) of SESN2 (p = 0.037). Unlike healthy cohorts, waist circumference (p <0.001), waist-to-hip ratio (p = 0.004), TyG x BMI (p < 0.028) TyG x waist circumference (p < 0.001), TyG x waist-to-hip ratio (p = 0.011) and LAP (p = 0.004) were significantly higher in the higher SESN2 tertiles (T2 and T3). This observation is counterintuitive and warrants further investigation to elucidate the underlying mechanisms. These results paint a picture of increased cardiometabolic risk associated with higher SESN2 levels in the diabetic cohort, which can be observed with a higher prevalence of MetS patients in the T3 group, although not statistically significant.

**Table 3:** Comparative analysis of cardiometabolic indices across tertile groups of SESN2 in the diabetic cohort.

Characteristic	Overall, N = 518	T1, N = 173	T2, N = 172	T3, N = 173	p-value
Gender					0.3
- Female	221 (43%)	79 (46%)	65 (38%)	77 (45%)	
- Male	297 (57%)	94 (54%)	107 (62%)	96 (55%)	
Age (years)	49.87 ± 11.20	49.36 ± 12.05	50.27 ± 10.50	49.98 ± 11.04	>0.9
BMI (kg/m²)	30.76 ± 5.56	29.94 ± 5.34	31.21 ± 5.81	31.13 ± 5.47	0.082
Biochemical Measurements					
SESN2 Concentration (ng/mL)	5.49 ± 5.94	1.59 ± 0.57	3.32 ± 0.58	11.54 ± 6.98	**<0.001**
Glucose (mmol/L)	9.80 ± 3.93	9.83 ± 3.92	9.71 ± 4.01	9.86 ± 3.88	0.8
Insulin (µIU/mL)	23.54 ± 52.21	25.06 ± 65.82	22.77 ± 41.30	22.79 ± 46.65	**0.008**
HbA1c (%)	8.41 ± 1.70	8.45 ± 1.66	8.42 ± 1.74	8.37 ± 1.72	0.7
Total Cholesterol (mmol/L)	4.79 ± 1.08	4.87 ± 1.15	4.73 ± 1.12	4.76 ± 0.96	0.6
HDL Cholesterol (mmol/L)	1.25 ± 0.35	1.29 ± 0.37	1.22 ± 0.30	1.24 ± 0.36	0.2
LDL Cholesterol (mmol/L)	2.77 ± 0.96	2.87 ± 1.02	2.72 ± 0.97	2.71 ± 0.88	0.3
Triglyceride (mmol/L)	1.75 ± 1.06	1.65 ± 0.95	1.78 ± 1.20	1.80 ± 1.03	0.2
Insulin Resistance and Adiposity					
Waist Size (cm)	97.83 ± 13.12	95.03 ± 12.41	99.15 ± 13.48	99.31 ± 13.08	**<0.001**
Waist-to-Hip Ratio	0.92 ± 0.09	0.90 ± 0.09	0.92 ± 0.09	0.93 ± 0.08	**0.004**
HOMA-IR	10.07 ± 20.01	9.94 ± 20.45	9.98 ± 17.84	10.27 ± 21.62	**0.037**
TyG Index	9.31 ± 0.70	9.26 ± 0.70	9.31 ± 0.68	9.36 ± 0.71	0.3
TG/HDL Ratio	1.58 ± 1.21	1.48 ± 1.13	1.60 ± 1.24	1.67 ± 1.25	0.14
TyG x BMI	285.95 ± 55.10	276.23 ± 51.26	290.05 ± 55.58	291.56 ± 57.30	**0.028**
TyG x Waist Circumference	910.67 ± 142.60	879.62 ± 135.77	921.44 ± 134.99	930.89 ± 151.84	**<0.001**
TyG x Waist-to-Hip Ratio	8.55 ± 1.11	8.38 ± 1.13	8.55 ± 1.04	8.71 ± 1.13	**0.011**
Visceral Adiposity Index (VAI)	2.41 ± 1.91	2.25 ± 1.67	2.38 ± 1.78	2.61 ± 2.24	0.12
Lipid Accumulation Product (LAP)	62.65 ± 42.40	55.63 ± 38.23	63.71 ± 43.49	68.59 ± 44.43	**0.004**
Atherogenic Index of Plasma (AIP)	0.10 ± 0.29	0.06 ± 0.31	0.11 ± 0.27	0.12 ± 0.30	0.14
Body Fat Percentage (%)	32.57 ± 10.45	31.17 ± 10.21	33.43 ± 10.79	33.15 ± 10.27	0.085
Blood Pressure (BP)					
Average Systolic BP (mmHg)	123.50 ± 14.63	121.47 ± 13.27	126.24 ± 15.74	122.79 ± 14.46	**0.017**
Average Diastolic BP (mmHg)	71.01 ± 10.15	70.64 ± 9.50	72.38 ± 10.66	70.01 ± 10.15	0.088
**Metabolic Syndrome (MetS)**	300 (58%)	92 (54%)	99 (58%)	109 (63%)	0.2
Condition1 (Central obesity)	410 (79%)	128 (74%)	139 (81%)	143 (83%)	0.11
Condition2 (High Triglyceride)	208 (40%)	60 (35%)	69 (40%)	79 (46%)	0.12
Condition3 (Low HDL)	206 (40%)	64 (37%)	68 (40%)	74 (43%)	0.6
Condition4 (High Glucose)	518 (100%)	173 (100%)	172 (100%)	173 (100%)	
Condition5 (High BP)	167 (32%)	52 (30%)	66 (38%)	49 (28%)	0.11

Values in bold indicate statistically significant results (p < 0.05).

### 3.3 Correlation analyses

Correlation analyses were conducted to investigate the relationship between SESN2 concentration and various clinical variables within the healthy and diabetic cohorts, respectively, and the results are illustrated in **Table 4**. These analyses aimed to uncover potential associations between SESN2 levels and a wide range of metabolic, adiposity, and blood pressure indices while controlling for the potential confounding effects of age, gender, and nationality. In the healthy cohort, there was a significantly negative correlation between insulin levels (r = -0.19, p = 0.0006) and SESN2 concentration and for HbA1c (r = -0.19, p = 0.0006). This intermediate approach suggests that a higher concentration of SESN2 may favorably influence glycemic control in healthy individuals. The negative correlation between SESN2 levels and HOMA-IR (r = -0.17, p = 0.0024), TG/HDL ratio (r = -0.12, p = 0.0283), TyG x BMI (r = -0.12, p = 0.0318), TyG x waist circumference (r = -0.11, p = 0.0466), VAI (r = -0.12, p = 0.0322), LAP (r = -0.12, p = 0.0320), AIP (r = -0.12, p = 0.0280) also reached statistical significance. These observations hint at a potential role of SESN2 in modulating glucose metabolism and adipogenicity.

**Table 4:** Correlation of SESN2 concentration with numerical variables (Adjusted for Age, Gender, and Nationality).

	Healthy		Diabetes	
	Correlation	P-value	Correlation	P-value
BMI	-0.09	0.1050	0.08	0.0573
Biochemical Measurements				
Glucose (mmol/L)	-0.01	0.9104	-0.02	0.6248
Insulin (µIU/mL)	-0.19	**0.0006**	0.15	**0.0005**
HbA1c (%)	-0.19	**0.0006**	-0.04	0.3091
Total Cholesterol (mmol/L)	0.09	0.1065	-0.01	0.7791
HDL Cholesterol (mmol/L)	0.09	0.0910	-0.07	0.0901
LDL Cholesterol (mmol/L)	0.10	0.0778	-0.05	0.2924
Triglyceride (mmol/L)	-0.10	0.0614	0.08	0.0619
Insulin Resistance and Adiposity				
Waist Size (cm)	-0.09	0.1193	0.15	**0.0006**
Waist-to-Hip Ratio	-0.07	0.2218	0.16	**0.0002**
HOMA-IR	-0.17	**0.0024**	0.11	**0.0106**
TyG Index	-0.09	0.1257	0.06	0.2065
TG/HDL Ratio	-0.12	**0.0283**	0.09	**0.0445**
TyG x BMI	-0.12	**0.0318**	0.10	**0.0215**
TyG x Waist Circumference	-0.11	**0.0466**	0.15	**0.0009**
TyG x Waist-to-Hip Ratio	-0.09	0.1015	0.12	**0.0060**
Visceral Adiposity Index (VAI)	-0.12	**0.0322**	0.09	**0.0358**
Lipid Accumulation Product (LAP)	-0.12	**0.0320**	0.14	**0.0011**
Atherogenic Index of Plasma (AIP)	-0.12	**0.0280**	0.09	**0.0446**
Body Fat Percentage (%)	-0.07	0.1842	0.11	**0.0170**
Blood Pressure (BP)				
Average Systolic BP (mmHg)	-0.08	0.1658	0.04	0.3124
Average Diastolic BP (mmHg)	0.04	0.5232	0.00	0.9868

Values in bold indicate statistically significant results (p < 0.05).

As in the healthy cohort, results from the diabetic cohort showed many correlation coefficients close to zero, indicating a lack of strong linear relationships between SESN2 and most of the numerical variables. In contrast to the healthy cohort, several significant positive correlations were observed in the diabetic group. SESN2 showed a positive correlation with waist circumference (r = 0.15, p = 0.0006), waist-to-hip ratio (r = 0.16, p = 0.0002), HOMA-IR (r = 0.11, p = 0.0106), TG/HDL ratio (r = 0.09, p = 0.0445), TyG x BMI (r = 0.10, p = 0.021), TyG x waist circumference (r = 0.15, p = 0.0009), TyG x waist to hip ratio (r = 0.12, p = 0.006), VAI (r = 0.09, p = 0.0358), LAP (r = 0.14, p = 0.0011), AIP (r = 0.09, p = 0.0446) and percentage body fat (r = 0.11, p = 0.0170). This suggests a potential link between higher SESN2 levels and increased adiposity and insulin resistance in individuals with diabetes. SESN2 also exhibited a significant positive correlation with insulin (r = 0.15, p = 0.0005) and triglycerides (r = 0.08, p = 0.0619). These findings further support the notion that SESN2 might be associated with metabolic dysregulation in the context of diabetes.

Comparing the correlation analyses between the two cohorts reveals intriguing differences. While a negative correlation was observed between SESN2 and HbA1c in the healthy cohort, no such relationship was evident in the diabetic group. Moreover, several positive correlations emerged between SESN2 and adiposity/insulin resistance indices in the diabetic cohort, while negative correlations were found in the healthy group. The contrasting findings might be attributed to underlying differences in the pathophysiology of glucose metabolism and insulin signaling between healthy individuals and those with diabetes. SESN2 may play distinct roles in these processes depending on the presence or absence of the disease.

### 3.4 Association analyses

The association of SESN2 concentration with various binary cardiometabolic outcomes in the healthy and diabetic cohort was estimated, and the results are presented in **Table 5**. Healthy individuals in the highest SESN2 tertile (T3) had significantly lower odds of high insulin (OR: 0.23, p < 0.01), high HbA1c (OR: 0.33, p < 0.01), high triglycerides (OR: 0.37, p = 0.03), high HOMA-IR (OR: 0.58, p = 0.06), high TyG x waist-to-hip ratio (OR: 0.39, p = 0.02), high VAI (OR: 0.46, p = 0.03), high LAP (OR: 0.49, p = 0.03), high AIP (OR: 0.42, p = 0.02) and the presence of MetS (OR: 0.23, p < 0.01). These findings indicate a potential protective role of SESN2 against insulin resistance, dyslipidemia, and MetS in healthy individuals. Conversely, in the diabetic cohort, higher SESN2 concentrations (T3) were associated with significantly higher odds of high triglycerides, high TyG x BMI, high TyG x waist circumference, high VAI, high LAP, high body fat, abdominal obesity, and high waist-to-hip ratio. This indicates that, in individuals with established diabetes, higher SESN2 levels might be linked to increased cardiometabolic risk factors. These contrasting associations between SESN2 and cardiometabolic parameters in the healthy and diabetic cohorts suggest that the role of SESN2 might be context-dependent and influenced by the presence or absence of diabetes. Further research is needed to elucidate the underlying mechanisms driving these differential associations and to explore the potential implications for targeted interventions aimed at modulating SESN2 levels in different populations.

**Table 5:** Association of SESN2 concentration (predictor) with binary variables (outcome) (Adjusted for Age, Gender, and Nationality).

Variables	Healthy				Diabetes			
	T2		T3		T2		T3	
	OR	p-value	OR	p-value	OR	p-value	OR	p-value
Overweight	0.77	0.42	0.82	0.56	1.86	0.07	1.96	**0.05**
High Glucose	1.34	0.51	1.66	0.25	0.82	0.43	0.96	0.88
High Insulin	0.59	0.18	0.23	**<0.01**	0.96	0.89	1.13	0.65
High HbA1c	0.30	**<0.01**	0.33	**<0.01**	1.07	0.78	0.84	0.46
High Total Cholesterol (TCH)	1.09	0.79	1.45	0.23	0.87	0.55	0.96	0.86
Low HDL	0.91	0.78	1.05	0.89	1.05	0.84	0.93	0.75
High LDL	1.28	0.40	1.51	0.17	0.70	0.11	0.72	0.13
High Triglycerides	0.82	0.62	0.37	**0.03**	1.23	0.36	1.57	**0.04**
High HOMA-IR	0.62	0.10	0.58	0.06	0.83	0.59	1.76	0.16
High TyG Index	1.36	0.29	1.60	0.14	1.26	0.80	0.68	0.62
High TG/HDL Ratio	0.36	0.38	0.39	0.42	1.12	0.77	1.22	0.59
High TyG x BMI	0.96	0.90	0.94	0.84	1.69	**0.02**	1.50	0.08
High TyG x Waist Circumference	0.53	0.08	0.79	0.51	1.88	**0.01**	1.80	**0.02**
High TyG x Waist-to-Hip Ratio	0.48	0.07	0.39	**0.02**	0.84	0.58	1.16	0.64
High VAI	0.66	0.16	0.46	**0.03**	1.31	0.22	1.54	**0.05**
High LAP	0.47	**0.03**	0.49	**0.03**	1.61	**0.03**	1.57	**0.04**
High AIP	0.69	0.30	0.42	**0.02**	1.28	0.26	1.38	0.14
High Body Fat	0.92	0.79	0.97	0.92	1.87	**0.01**	1.72	**0.02**
Abdominal Obesity (Waist Size)	0.67	0.19	0.95	0.86	1.62	**0.08**	1.82	**0.03**
High Waist-to-Hip Ratio	0.56	0.11	0.68	0.28	0.87	0.58	1.96	**0.01**
High Average Systolic BP	0.99	0.99	0.72	0.37	1.75	**0.02**	1.21	0.40
High Average Diastolic BP	0.44	0.10	1.33	0.51	1.28	0.34	0.91	0.74
Metabolic Syndrome (MetS)	0.25	**<0.01**	0.23	**<0.01**	1.25	0.32	1.48	**0.08**

Values in bold indicate statistically significant results (p < 0.05).

In summary, lower SESN2 concentrations were significantly associated with a higher prevalence of MetS. Compared to the lowest tertile (T1), individuals in the middle (T2) and highest (T3) tertiles of SESN2 levels had lower odds of MetS (T2 OR: 0.25, p < 0.01; T3 OR: 0.23, p<0.01). Higher SESN2 levels were associated with better glycemic control. Individuals in T2 and T3 had significantly lower odds of elevated HbA1c (T2 OR: 0.30, p < 0.01; T3 OR: 0.33, p < 0.01) compared to T1. Higher SESN2 levels were associated with lower insulin resistance. The odds of having high HOMA-IR were reduced in T3 although not statistically significant (OR: 0.58, p = 0.06) compared to T1. Individuals in the highest tertile of SESN2 had significantly lower odds of high triglycerides (T3 OR: 0.37, p = 0.03) compared to T1. Higher SESN2 levels were associated with more favorable adiposity profiles. Individuals in T3 had significantly lower odds of high TyG x Waist-to-Hip Ratio (OR: 0.39, p = 0.02), high VAI (OR: 0.46, p = 0.03), high LAP (OR: 0.49, p = 0.03), and high AIP (OR: 0.42, p = 0.02) compared to T1. In contrast to the healthy cohort, higher SESN2 levels in the diabetic group were associated with increased adiposity. Individuals in T3 had higher odds of being overweight (OR: 1.96, p = 0.05) and having abdominal obesity (OR: 1.82, p = 0.03) compared to T1. Higher SESN2 levels were associated with increased odds of high triglycerides (T3 OR: 1.57, p = 0.04) in the diabetic group. Higher SESN2 levels were associated with unfavorable adiposity profiles in diabetics. T3 had higher odds of elevated TyG x Waist Circumference (OR: 1.80, p = 0.02), VAI (OR: 1.54, p = 0.05), LAP (OR: 1.57, p = 0.04), and body fat percentage (OR: 1.72, p = 0.02) compared to T1. Interestingly, individuals in T2 had higher odds of elevated systolic blood pressure (OR: 1.75, p = 0.02) compared to T1, but this association was not observed in T3.

## 4 Discussion

The present study explored the association of plasma levels of SESN2 with adiposity and metabolic indices in both healthy and diabetic subjects from QBB. SESN2, a stress-inducible protein, plays a significant role in the regulation of oxidative and metabolic stress, which are critical factors in the development and progression of cardiometabolic diseases (26). Our findings show that SESN2 levels are significantly reduced in subjects with diabetes compared to healthy individuals, suggesting a preventive role of SESN2 in such conditions. This reduction could affect the body’s response to increased oxidative and ER stress commonly observed in metabolic disorders (27). ER stress due to higher protein synthesis activity in metabolic disorders like obesity and diabetes is a converging link between oxidative stress, insulin resistance, and endothelial dysfunction (28). We have previously shown that suppression of SESN2 can aggravate oxidative stress in response to pharmacological induction of ER stress (29). Previous studies have demonstrated that SESN2 regulates the balance of antioxidant enzymes and mitigates oxidative stress, contributing to cellular protection and improved metabolic function. Moreover, SESN2 has been shown to activate AMP-activated protein kinase (AMPK), which in turn induces anti-inflammatory and antioxidative responses, highlighting its potential role in managing metabolic disturbances (27, 30). The observed decrease in SESN2 levels among diabetic subjects in our study aligns with earlier research indicating that SESN2 down-regulation could result in metabolic stress and conditions such as obesity and insulin resistance (15). In experimental models, lack of SESN2 has been associated with increased progression of diabetes and obesity-induced insulin resistance, highlighting its protective role in metabolic stress conditions (31). Several mechanisms could explain the differential associations of SESN2 with cardiometabolic parameters in healthy and diabetic individuals. SESN2 is involved in various cellular processes, including antioxidant defense, autophagy, and regulation of metabolism (4). With the progression of the disease, the compensatory mechanism no longer keeps up with the stress, and the protective mechanisms fall apart (8). The genetic predisposition to reduced levels of SESN2 cannot be ruled out as well in certain individuals, exposing them to higher odds of developing or progression of the disease.

Our study provides a comprehensive analysis of the relationship between SESN2 levels and cardiometabolic parameters in healthy and diabetic individuals. In the healthy cohort, we found that HbA1c, HOMA-IR, insulin, and glucose levels are lower in higher tertiles of SESN2, indicating a protective role of SESN2. This is further supported by the observation that SESN2 levels were negatively correlated with HbA1c, and insulin levels and individuals categorized in T2 and T3 have lower odds of high HbA1c, and insulin as compared to T1. The percentage of individuals with high adipogenicity indices like VAI, LAP, AIP, and TyG x waist-to-hip ratio was also lower in individuals falling under the higher tertile of SESN2. As all these factors contribute to MetS, there was a lower percentage of patients with MetS in T2 and T3 of SESN2 when compared with T1. These findings align with previous studies reporting the beneficial effects of SESN2 on glucose metabolism and insulin sensitivity (13, 14). However, the relationship between SESN2 and cardiometabolic parameters appears to be more complex in the diabetic cohort. SESN2 levels were lower in the diabetic cohort when compared with the healthy cohort. This aligns with previous studies where Sesn2 levels were found to be lower in diabetic individuals (32). While we did not observe a significant correlation between SESN2 and HbA1c, SESN2 levels had a positive correlation with insulin, HOMA-IR, and the majority of the obesity and adipogenicity indices. This suggests that, in the context of established diabetes, higher SESN2 levels might be linked to increased cardiometabolic risk. It completely aligns with the findings of Chung et al. where a positive correlation between SESN2 levels and BMI, waist circumference, waist-to-hip ratio, and HOMA-IR was observed (13) but contrasts with some other studies where a negative correlation between SESN2 levels and markers of insulin resistance and adipogenicity in diabetic patients was found suggesting a protective role of SESN2 in diabetes (32). An increase in SESN2 levels with high adiposity also aligns with others, suggesting a potential contribution of SESN2 to disease progression (14). A complex interplay between obesity and diabetes may exist in the diabetic cohort, and the smaller negative correlations are overshadowed by the slight increase in SESN2 levels due to these comorbidities.

Despite these promising findings, our study has some limitations. First, the cross-sectional nature of the data limits our ability to infer causality between SESN2 levels and cardiometabolic indices, and residual confounding cannot be entirely ruled out, even after adjustment for covariates. Longitudinal studies are needed to establish a temporal relationship and to better understand the mechanisms underlying SESN2’s protective effects. Second, our study population was limited to subjects from QBB, and the findings may not be generalizable to other populations with different genetic and environmental backgrounds. Third, the diabetic population in our study was, on average, older than the healthy control group. This age difference could potentially have confounded the observed associations. To address this, we adjusted for age in all association models. However, while this adjustment significantly reduces the potential for confounding by age, we acknowledge that residual confounding by age, or by factors closely correlated with age, cannot be completely ruled out. As far as medication use as a confounding variable is concerned, 95.9% of the diabetic cohort was on anti-diabetic medication, and 14.1% of the diabetic cohort was on statin medication at the time of enrolment in the study. While we adjusted our primary association models for age, sex, and nationality, we did not perform a subgroup analysis specifically stratifying by medication use (anti-diabetic or statin) within the diabetic cohort due to relatively small number of individuals on statins within the diabetic cohort (14.1%) which would have limited the statistical power of such a subgroup analysis. Future research with more comprehensive medication data, including drug types, dosages, and durations of use, is needed to fully understand the effects of SESN2, specific medications, and cardiometabolic health in individuals with diabetes. This includes conducting appropriately powered subgroup analyses to examine medication-specific effects.

Nevertheless, our results provide valuable insights into the complex relationship between SESN2 and cardiometabolic health and disease, highlighting the need for further research to elucidate the underlying mechanisms and the potential implications for targeted interventions. The study has several strengths, including the use of a large, well-characterized cohort from a population-based biobank, the rigorous measurement of SESN2 and other clinical parameters, and the application of appropriate statistical methods.

## 5 Conclusions

In conclusion, these results suggest that the relationship between SESN2 levels and metabolic health indicators differs between healthy individuals and those with diabetes. In the healthy cohort, higher SESN2 levels were generally associated with a more favorable metabolic profile, while in the diabetic cohort, higher SESN2 levels were associated with increased adiposity and some unfavorable metabolic indicators. These findings highlight the complex role of SESN2 in metabolic regulation and suggest that its effects may be context-dependent.

## Data Availability

The original contributions presented in the study are included in the article/supplementary material. Further inquiries can be directed to the corresponding author.
